# Suspected geriatric onset of attention-deficit/hyperactivity disorder in a patient with comorbid bipolar disorder

**DOI:** 10.1186/s12877-024-04852-2

**Published:** 2024-03-05

**Authors:** Joshua Woods, Nakisa Kiai, Ranjit Padhy, Rossano Bangasan, Tyler Torrico

**Affiliations:** 1 Ross University School of Medicine, Miramar, Florida USA; 2 Kern Medical, Department of Psychiatry, Bakersfield, California USA

**Keywords:** ADHD, Psychostimulants, Geriatric psychiatry, Bipolar disorder

## Abstract

**Background:**

An increasing number of adults over 60 years old are presenting with requests for treatment of attention-deficit/hyperactivity disorder (ADHD). However, the prevalence of ADHD in older adults in geriatrics is unknown. Further, comorbid bipolar disorder and adult ADHD are likely underrecognized with many patients only receiving treatment for one of these conditions. The occurrence of bipolar disorder with geriatric onset ADHD is unknown.

**Case presentation:**

A 64-year-old Hispanic woman with a psychiatric history of bipolar I disorder (diagnosed in early adulthood) was diagnosed with ADHD suspected of geriatric onset, and able to be successfully managed on concurrent mood stabilizers and psychostimulant medication.

**Conclusions:**

The findings of this case report emphasize the importance of appropriately recognizing and treating comorbid ADHD and bipolar disorder in any age group, including the geriatric population for which this occurrence appears to be very rare. Additionally, this case report demonstrates the safe utilization of psychostimulant medications in a geriatric patient with bipolar disorder without inducing a manic episode or other significant adverse reactions.

## Background

An increasing number of adults over 50 years old are presenting with requests for treatment of attention-deficit/hyperactivity disorder (ADHD) [1]. However, the exact prevalence of ADHD in older adults in geriatrics is unknown. The prevalence of ADHD is generally accepted among psychiatrists to decrease with age, but there are limited controlled studies that investigate using ADHD medications in adults over 50 years old [1]. There are estimates that childhood ADHD may persist into adulthood at age 25 between 15 and 65% of cases [2]; however, older adults and geriatrics with ADHD are an even less studied population with estimates generally below 5% of the population [3, 4].

Although ADHD and bipolar disorders are common mental health conditions, the degree of their comorbid occurrence in adulthood, and especially geriatric age, remains unclear. There are estimates of ADHD and adult bipolar disorder occurring comorbidly in as many as 1 in 13 patients; however, many of these cases may be unrecognized and untreated [5]. Other studies report even higher comorbidity, emphasizing the need for accurate diagnosis and treatment plan formulation [6]. The under-recognition of both disorders when they occur comorbidly is likely due to some symptomatology overlap, as well as the similar impacts that each condition has on major life area functioning for patients [7]. Still, appropriate recognition and treatment of both conditions are optimal, and the effective management of both conditions is likely to result in improved quality of life and functioning [1, 7].

## Case presentation

A 64-year-old Hispanic woman with a psychiatric history of bipolar I disorder (diagnosed in early adulthood) presented in the outpatient setting for the continuation of psychiatric care after moving to the area. Her prior uncontrolled symptoms included pressured speech, rapid ideas of importance, and insomnia. She also reported new short-term memory problems. Her psychiatric management included amitriptyline (25 mg daily), valproic acid extended release (1500 mg nightly), and quetiapine (50 mg twice daily).

On continued follow-up, she complained of forgetfulness and dropping items which led to feelings of sadness and frustration. A neurologic exam revealed no focal deficits at that time. She was prescribed donepezil (titrated to 10 mg daily) for treatment of possible dementia. Over the following year, her symptoms continued, and the patient relapsed into a major depressive episode. Neurology was consulted for continued concerns of dementia, with laboratory studies returning within normal limits (TSH, T4, T3 total, erythrocyte sedimentation rate, c-reactive protein, vitamin B12, RPR). Electroencephalogram (EEG) demonstrated mild generalized slowing, with no focal slowing or seizure activity. Computed tomography (CT) scan and magnetic resonance imaging (MRI) were unremarkable. Neurology recommended discontinuing donepezil and amitriptyline out of concern for medication adverse reactions and concerns of pseudodementia.

The patient was monitored over the following year for alleviation of symptoms but did not have significant change despite her medication changes. She continued to complain of difficulty with concentration, memory, and restlessness but did have some improved mood. Due to the persistence of forgetfulness, ADHD was considered in the differential diagnosis. Her evaluation for ADHD at this time was consistent with many DSM-5 criteria for inattention including difficulty sustaining attention, frequent careless mistakes, not following through on instructions, frequently losing necessary items, difficulty maintaining sequential tasks, being easily distracted, and did not listen when spoken to directly as if her mind was elsewhere.

The patient was prescribed methylphenidate 5 mg twice a day and reported near-immediate improved focus, increased energy, and improvement in her memory. 6 months into treatment the patient developed insomnia which resolved by decreasing her dose of methylphenidate to 5 mg daily. However, 4 months after the decrease in dose, symptoms of inattention returned, and she was restored to methylphenidate 5 mg twice daily and tolerated the medication without insomnia. The patient was followed for 3 years thereafter before moving out of the county, during this time she had no reported adverse reactions to methylphenidate and continued alleviation of symptoms.

## Discussion

This case report describes a suspected rare onset of geriatric ADHD in the context of long-standing adult bipolar disorder that was effectively managed with psychostimulant medication and mood stabilizers. A limitation to this case study includes the limited psychiatric history of symptoms prior to her presentation in our care system, and it is unclear how long she had been suffering from symptoms of ADHD; as well as the lack of symptom scoring data. It is possible that ADHD could have onset at a younger age and been underdiagnosed, but the patients presenting chief complaint of new forgetfulness suggests geriatric onset. Additionally, methylphenidates’ possible antidepressant effect may also have therapeutic benefit in this case. The findings of this case report emphasize the importance of appropriately recognizing and treating suspected comorbid ADHD and bipolar disorder in any age group, including the geriatric population for which this occurrence appears very rare, as there are no known reported cases prior.

Importantly, major neurocognitive disorders were considered in the differential diagnosis for this patient, and donepezil was prescribed by psychiatry prior to the initiation of psychostimulants with no observed clinical benefit, although this does not rule out a possible neurocognitive diagnosis. Additionally, neurology consultation was not suggestive of cognitive decline, but rather of suspected undertreated psychiatric illness, and even recommendation to discontinue donepezil. The incidence of major and minor neurocognitive disorders is far more common in the geriatric population than geriatric onset ADHD. Still, both conditions also have overlapping symptoms in regard to difficulties with concentration and attention. Notably, there have been recent investigations of utilizing psychostimulants (including methylphenidate) in treating apathy associated with Alzheimer’s disease [8, 9].

Methylphenidate is also used off-label for major depression in the elderly with medical illness, palliative care, or terminal illness [10]. It is possible that the patient we describe in this case report benefitted from methylphenidate in this manner, reducing symptoms of possible pseudodementia. However, because the primary symptoms were intention with subsequent frustration and depression thereafter, we consider it more likely that the therapeutic response was due to treating the geriatric onset ADHD. Although, if pseudodementia was the primary concern, this case would still demonstrate a safe and effective utilization of methylphenidate in an elderly patient, and there is evidence that psychostimulant augmentation of antidepressants may be an effective strategy in adults [11].

Utilizing psychostimulants with other psychotropic medications in the geriatric population requires careful monitoring, as they may be more prone to adverse reactions even from lower doses [12]. Further, some clinicians may have hesitations about prescribing stimulant medications for ADHD to those with comorbid bipolar disorder or a history of substance use disorders [7]. These hesitations may be mitigated with proper risk management, including assessment for cardiac tolerability, monitoring for the development of mania/hypomania, and utilizing controlled substance review programs [4].

## Conclusions

The findings of this case report emphasize the importance of appropriately recognizing and treating suspected comorbid ADHD and bipolar disorder in any age group, including the geriatric population for which this occurrence is very rare. Additionally, this case report demonstrates the safe utilization of psychostimulant medications in a geriatric patient with bipolar disorder without inducing a manic episode or other significant adverse reactions.

## Data Availability

No datasets were used or generated for this manuscript. All data for the case report is included in the published article.

## Abbreviations

ADHD: attention-deficit/hyperactivity disorder
TSH: thyroid stimulating hormone
RPR: rapid plasma reagin
EEG: Electroencephalogram
CT: Computed tomography
MRI: magnetic resonance imaging
DSM-5: the diagnostic and statistical manual of mental disorders, fifth edition
